# Extrathyroidal Manifestations of Persistent Sporadic Non-Autoimmune Hyperthyroidism in a 6-Year-Old Boy: A Case Report

**DOI:** 10.3390/life11070713

**Published:** 2021-07-19

**Authors:** Moon Bae Ahn

**Affiliations:** Department of Pediatrics, College of Medicine, Catholic University of Korea, 222 Banpo-daero, Seocho-gu, Seoul 06591, Korea; mbahn@catholic.ac.kr; Tel.: +82-2-2258-6756

**Keywords:** congenital hyperthyroidism, thyrotropin receptors, precocious puberty, intraventricular hemorrhage, bladder stones

## Abstract

Thyroid-stimulating hormone receptor (TSHR) belongs in a subfamily of the G protein-coupled receptors. Thyroid-stimulating hormone receptor gene (*TSHR*), a gene encoding TSHR, is a major controller of thyroid cell metabolism, and its gain of function mutation leads to non-autoimmune hyperthyroidism (NAH), a condition of a prolonged state of hyperthyroidism. Diverse human diseases, and genetic, constitutional, or environmental factors contribute to the phenotypic variations of *TSHR* mutations; however, the underlying mechanisms leading to various extrathyroidal manifestations across ages are poorly understood. In 2018, the first Korean case of persistent sporadic NAH due to missense mutation of *TSHR* was reported, and this report highlights the extrathyroidal manifestations of NAH. Further investigation is warranted to clarify the roles of functional mutations of TSHR by investigating the correlation between G protein-dependent signaling properties and clinical phenotypes associated with persistent hyperthyroidism in order to develop novel therapies that could be provided for numerous conditions caused by NAH.

## 1. Introduction

Congenital hyperthyroidism (CH) is a condition characterized by excessive production of thyroid hormone soon after birth. The most common cause of CH is Graves’ disease, which occurs as a result of placental passage of maternal thyroid hormone receptor-stimulating antibodies to the newborn [1]. This type of autoimmune hyperthyroidism resolves spontaneously 3 to 12 weeks after birth when maternal antibodies are eliminated from an infant’s blood [2]. On the other hand, non-autoimmune hyperthyroidism (NAH) is a less common form of childhood hyperthyroidism that occurs as a result of a mutation of the thyroid-stimulating hormone receptor gene (*TSHR*) [3]. NAH may occur as a result of McCune–Albright syndrome, a condition caused by a postzygotic, somatic, activating mutation of the α-subunit of the G-protein with additional findings of gonadotropin-independent precocious puberty, café-au-lait macules, and fibrous dysplasia [1,4,5]. These types of neonatal hyperthyroidism cases are permanent. However, the use of antithyroid drugs to treat this type of hyperthyroidism is limited, and normal thyroid function can only be achieved by complete ablation of the thyroid tissue [6].

Thyroid-stimulating hormone receptor (TSHR) is located on chromosome 14q31 and belongs in a subfamily of the G protein-coupled receptors (GPCRs) [3]. Increased growth of the thyroid follicular cells and hormonogenesis are stimulated by the activation of adenylyl cyclase, and gain-of-function (GoF) mutations of THSR lead to a prolonged state of hyperthyroidism, resulting in the constitutive binding of thyrotropin (thyroid-stimulating hormone [TSH]) to TSHR [7]. According to available data, 29 *TSHR* disease-causing variants have been identified for familial NAH characterized by autosomal dominant inheritance, and 41 families have been reported [3,8]. Less than 20 patients with 13 activating de novo mutations of THSR regarding persistent sporadic NAH have been published [9].

Mutations of GPCR are associated with diverse human diseases, and genetic, constitutional, or environmental factors contribute to the phenotypic variations of *TSHR* mutations [10]. Intrauterine growth retardation, premature birth, craniosynostosis, developmental impairment, jaundice, hepatosplenomegaly, thrombocytopenia, and respiratory and neuromuscular symptoms are commonly presenting features in children with NAH, while precocious puberty and short stature are possible endocrinologic sequelae in patients whose proper management is delayed [6]. Although specific G protein-dependent endogenous signal pathways have been discovered that explain the pathogenesis of NAH due to *TSHR* GoF mutation, the underlying mechanisms leading to various extrathyroidal manifestations across ages are poorly understood.

In 2018, the first Korean case of persistent sporadic NAH due to missense mutation of *TSHR* (c.1899C>A, [p. Asp633Glu]) was reported [9]. The patient has been maintaining a euthyroid state while titrating the dosage of an antithyroid drug; thus, thyroidectomy has not been performed until recently. We have revisited the case and have reported extrathyroidal manifestations in a 6-year-old boy with persistent sporadic NAH.

## 2. Case Report

The patient was delivered at 33 weeks of gestation by emergent cesarean section owing to fetal tachycardia (185 beats/min) and was small for gestational age, with a birth weight of 2280 g (50–75th centiles), height of 43 cm (10–25th centiles), and head circumference of 31 cm (50–75th centiles). Immediate intubation followed by mechanical ventilation was commenced owing to meconium aspiration syndrome. His heart rate did not fall during ventilator care, and he remained tachycardic even after he was extubated. The thyroid function test at 4 days after birth revealed a marked elevation of free thyroxine (3.98 ng/dL) and total triiodothyronine (10.52 ng/mL) concentrations, with decreased thyrotropin (0.05 mIU/L) concentration in the absence of antibodies against thyrotropin receptor, thyroid peroxidase, and thyroglobulin. Thyroid ultrasound and technetium-99m scan revealed increased vascularity and uptake, respectively, with the enlargement of both glands. Propylthiouracil (5 mg/kg) was started and replaced with methimazole (0.5 mg/kg) at 6 months of age, while the patient’s euthyroid state was maintained. Hyperthyroidism was relapsed after tapering the dosage of the antithyroid drug; thus, treatment with methimazole was continued. A de novo heterozygous missense mutation of c.1899C>A (p.Asp633Glu) in exon 10 of *TSHR* was confirmed by Sanger sequencing and was the only possible explanation for the constitutive hypersecretion of the thyroid hormone.

**Figure 1:**
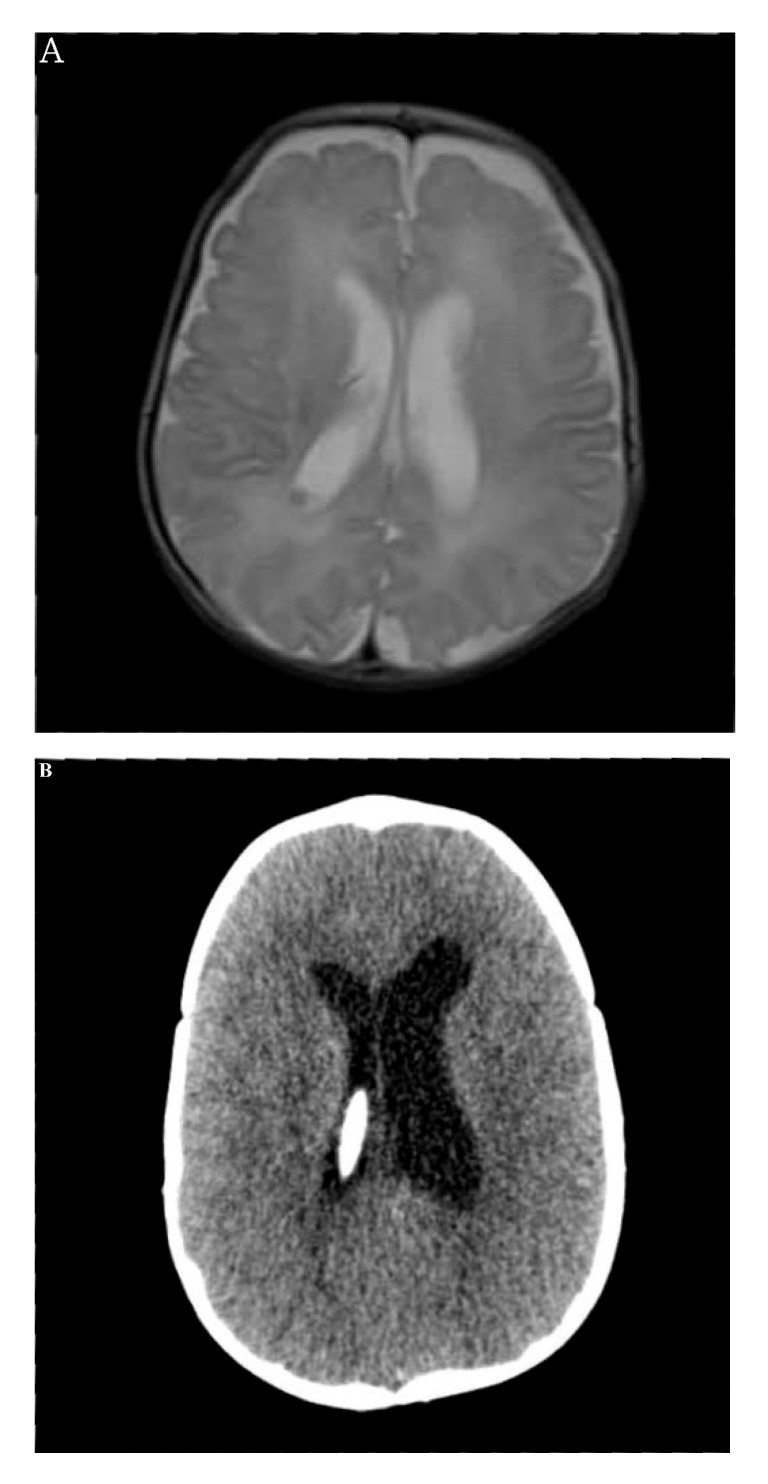
Neurologic manifestation associated with non-autoimmune hyperthyroidism: Ventriculomegaly. (**A**) Brain magnetic resonance imaging at 1 month of age showing both mildly dilated lateral ventricles and intraventricular hemorrhage in the right lateral ventricle (grade 3). (**B**) Computed tomography at 4 months of age showing ventriculoperitoneal shunt insertion into the right lateral ventricle.

At 1 month of age, bilateral dilation of the ventricles was noted by routine premature examination of brain ultrasound. Brain magnetic resonance imaging (MRI) revealed grade 3 intraventricular hemorrhage (IVH) in the right lateral ventricle, with mild dilation of both lateral ventricles (Figure 1A) [11].

Free thyroxine and total triiodothyronine levels were decreased yet elevated, while thyrotropin concentration remained low (Table 1).

Three-dimensional computed tomography detected intact cranial sutures with open anterior and posterior fontanelles. Ventriculomegaly was rapidly aggravated after 3 months of age, and the patient underwent ventriculoperitoneal shunt operation (Figure 1B). The insertion of a ventriculoperitoneal shunt led to partial regression and decompression of ventriculomegaly; nevertheless, a third ventriculostomy was inevitable when ventriculomegaly recurred at 17 months of age. Developmental delay was observed in all milestones, including cognitive skills, receptive-expressive communication, and gross-fine motor skills. Preschool psychological test at 6 years revealed full-scale intelligence quotient of 65 and social maturity scale social quotient of 62, indicating mild and educable intellectual disability.

**Figure 2:**
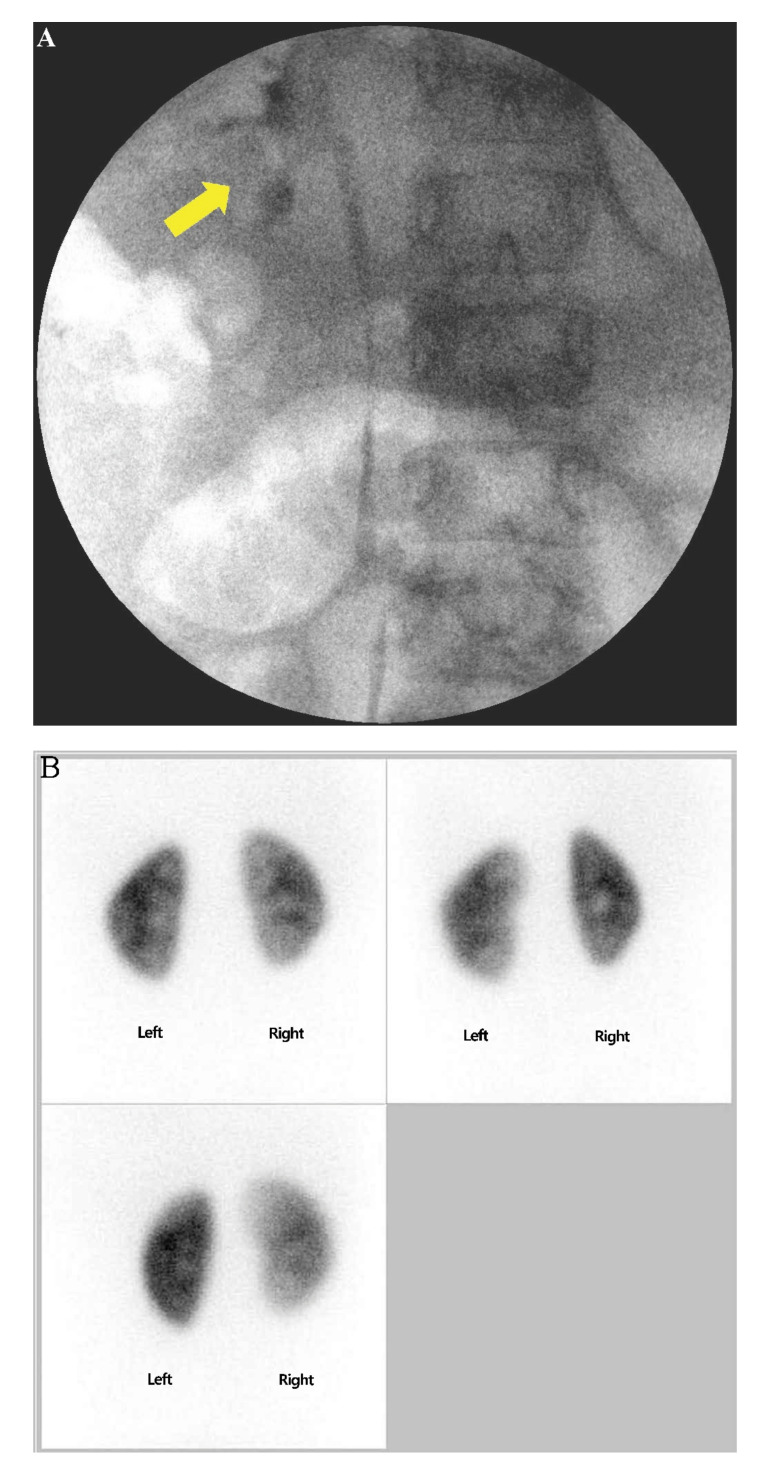

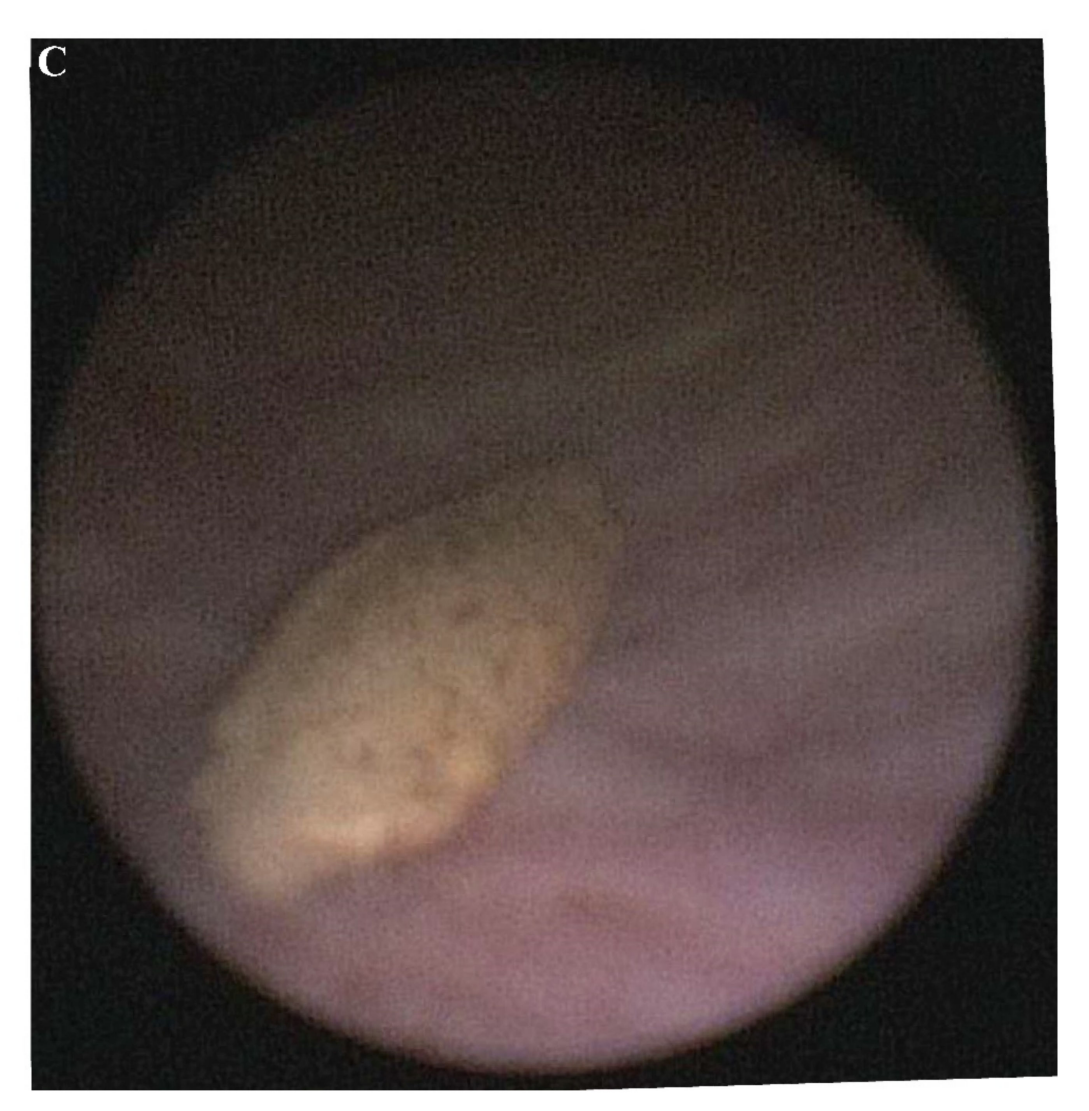
Urinary manifestation associated with non-autoimmune hyperthyroidism: Vesicoureteral reflux and urinary tract infection. (**A**) Voiding cystourethrography showing contrast reflux with mild to moderate dilation (yellow arrow) of the right ureter, renal pelvis, and calyces, indicating grade 3 vesicoureteral reflux. (**B**) ^99m^Tc-Dimercaptosuccinic acid scintigraphy cortical scan showing a decreased uptake in upper and lower portions of the right kidney, indicating acute pyelonephritis. (**C**) A 1.7-cm bladder stone detected using a cystoscope; cystolitholapaxy was performed for its removal.

At 26 months of age, the patient visited an emergency department due to irritability caused by dysuria and lower abdominal pain. Urinary white blood cell (WBC) count and calcium to creatinine ratio (UCa/Cr) were slightly elevated by 4–9 per high-power field (HPF) and 0.36, respectively. Hematuria, ketonuria, glycosuria, and proteinuria were not observed, and the patient’s vital sign was stable. No elevation of serum WBC count and C-reactive protein level was observed. Thyroid function was stable (Table 1). Ultrasound revealed normal renal parenchyma with a mildly thickened bladder wall. The initial urine culture result was positive (105 colony-forming units per mL) for enteric Gram-negative rods, which were sensitive to aminoglycosides. Intravenous amikacin was continued for 4 days until the negative conversion. Dysuria was resolved, and the patient was discharged. At 35 months of age, the patient revisited the emergency department with the same complaints. Gross hematuria (urinary red blood cell counts > 100 per HPF) and elevated serum calcium level (10.7 mg/dL) were newly noted, while hypercalciuria progressed by a UCa/Cr of 0.79. Serum phosphorus, parathyroid hormone, and bone-specific alkaline phosphatase levels were normal. The patient’s urinary WBC count was 4–9 per HPF with no microorganism cultured. Voiding cystourethrography revealed grade 3 right vesicoureteral reflux, and 99mTc-dimercaptosuccinic acid cortical scan detected acute pyelonephritis (Figure 2A,B).

A cystoscope was introduced to examine the whole bladder, revealing a 1.7-cm-sized stone; therefore, endoscopic cystolitholapaxy was performed (Figure 2C). Endoscopic subureteral polydimethylsiloxane injection was performed for the management of right vesicoureteral reflux. The patient was discharged without postoperative complications, and no further episodes of hypercalciuria, pyuria, and dysuria were observed.

**Figure 3:**
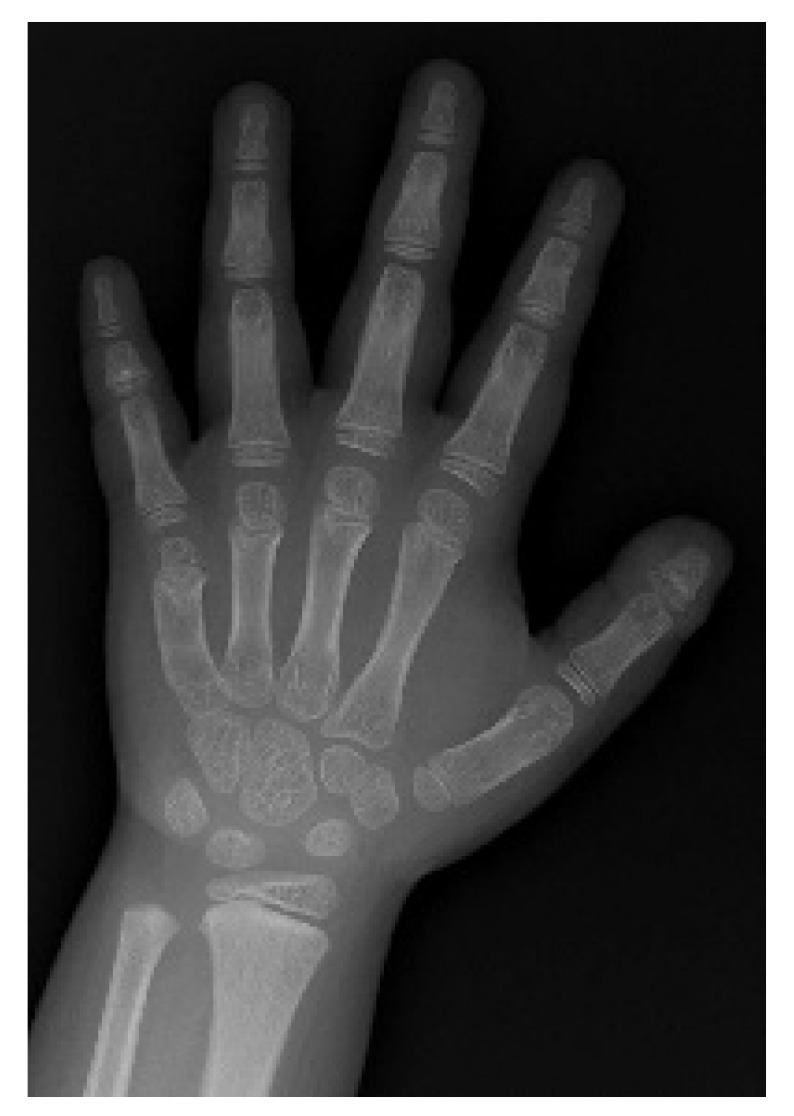
Endocrinologic manifestation associated with non-autoimmune hyperthyroidism: the patient showed bone age advancement of 2 years (Bone age was 89 months at chronological age of 63 months), which indicated precocious puberty evidenced by further acceleration.

The patient was growing normally until 36 months of age, and his height and weight were consistently reaching above the 75th percentile starting 48 months of age. At 63 months of age, a 4-mL testicular volume and phallic enlargement were observed, while his height and weight were 115.2 cm (75–90th percentiles) and 21.7 kg (75–90th percentiles), respectively [12]. Café-au-lait spots were not observed. The patient’s bone age showed further acceleration when compared with previous radiographs at age of 12 months (Figure 3) [9].

Baseline hormonal studies showed normal range for a prepubertal boy, while the subsequent luteinizing hormone (LH)-releasing hormone stimulation test (0.1 mg, gonadorelin) revealed a peak LH level of 4.1 mIU/mL and a peak follicular-stimulating hormone level of 5.2 mIU/mL. These findings were consistent with gonadotropin-dependent precocious puberty (GDPP), and treatment with gonadotropin-releasing hormone analog (GnRHa) was initiated. Sella MRI revealed a normal structure of the pituitary gland. Thyroid function was unremarkable (Table 1). After 6 months of treatment, post-GnRHa LH level showed adequate suppression of the hypothalamic-pituitary-gonadal axis.

## 3. Discussion

GPCRs are the largest family of cell surface proteins encoded by the human genome and account for many physiological functions by binding different types of ligands. TSH binding to TSHR is critical for growth and function of the thyroid gland and production of thyroid hormones [11]. The TSHR is a member of the glycoprotein-hormone receptor subfamily of family A GPCRs, and it plays a critical role in the endocrine signaling cascade, which activates both Gs/cAMP and Gq/phospholipase C/inositol phosphates/Ca^2+^ in human thyroid cells [13,14]. *TSHR*, a gene encoding TSHR, is a major controller of thyroid cell metabolism, and its loss or GoF mutation may lead to either CH, resulting in persistent hyperproduction of thyroid hormones, or congenital hypothyroidism by complete or partial TSH resistance with a thyroid phenotype from an apparent athyreosis to the gland in situ [6,15]. This case report presents a typical case of NAH due to GoF mutation of *TSHR*, with the patient eventually requiring surgical ablation of the whole thyroid. Nevertheless, until recently, maintaining a euthyroid state for this patient could have been possibly achieved by meticulous monitoring and administering methimazole with dose adjustments. To the best of our knowledge, this is the first case documenting three different extrathyroidal manifestations in a patient with NAH.

Thyroid hormones have important roles in embryogenesis and fetal maturation, and low birth weight and prematurity are the independent consequences observed in children with NAH [16]. Prolonged hyperthyroidism during the intrauterine period leads to fetal tachycardia, intrauterine growth retardation, and accelerated bone maturation and is a major trigger of premature birth [17]. Craniosynostosis is a common craniofacial manifestation in children with NAH, since osteoblastic bone formation and terminal hypertrophic differentiation in chondrocytes are accelerated by excessive thyroid hormone [18]. Nevertheless, the aggravation of ventriculomegaly was considered the outcome not of craniosynostosis but of IVH complicated by prematurity. Ventriculomegaly could have also been observed as a result of the thinning of the brain parenchyma due to the excessive production of thyroid hormones [19]. It was interesting to note that premature fusion of the calvarial sutures did not occur although an overtly hyperthyroid state persisted during the first 12 months after birth. In addition, hypothyroidism of prematurity, a possible consequence observed as a result of the immature hypothalamic-pituitary-thyroidal axis, could have been masked by over-hyperthyroidism. Neurosurgical intervention seems inevitable in children with NAH not only owing to craniosynostosis but also owing to prematurity, which is another risk factor for IVH followed by ventriculomegaly. For long-term management, developmental assessment should not be delayed in children with NAH, since neurological disorders may result in neurocognitive impairment and intellectual disability [20].

Although hypercalcemia is a rare presentation of hyperthyroidism, several adult cases of hyperthyroidism-associated hypercalcemia have been reported [21,22]. Its pathophysiologic mechanism is poorly understood; however, previous studies have suggested that the thyroid hormone might affect the calcium metabolism by altering bone formation and resorption via increased bone turnover and dysregulated osteoclastic activity [21,23]. Likewise, urinary stone formation in children with NAH might have been affected by hyperthyroidism-induced hypercalcemia. The bladder stone was strongly considered the cause of dysuria since there was no evidence of urinary tract infection on his revisit at 35 months and pain was dramatically reduced after stone removal. The calcium-sensing receptor, a family C GPCR, allows the regulation of parathyroid hormone secretion and renal tubular calcium reabsorption in response to altering extracellular calcium concentrations [24]. Its loss-of-function mutations is associated lifelong dysregulation of extracellular calcium levels, such as familial hypocalciuric hypercalcemia and primary hyperparathyroidism [25]. However, the patient was negative for calcium-sensing receptor gene mutation, while his parathyroid hormone levels were normal. Although a direct cause–effect of *TSHR* mutation on calcium homeostasis has not been clarified, prolonged hyperthyroid state was more likely responsible for stone formation regardless of maintaining a normal thyroid function after antithyroid drug therapy.

In the hyperthyroid state, pubertal onset and progression may be either advanced or delayed. Bone age is mostly advanced at the time of diagnosis of NAH, and the risk of precocious puberty might complicate the disease course in patients who are at a later stage of the disease and have delayed the start of treatment [6]. In McCune–Albright syndrome, hyperthyroidism is a classical phenotype associated with a higher number of endocrinopathies, including gonadotropin-independent precocious puberty [26]. Post-stimulated LH level did not increase above 5.0 mIU/mL, which was the cut-off according to the Korean diagnostic criteria of GDPP. However, mildly elevated basal LH concentration and significant increment after LH-releasing hormone stimulation were consistent with the finding of GDPP [27]. GnRHa treatment was necessary to slow down the rapid pubertal progression and bone age advancement. GoF GPCR mutations such as KISS1R encoding GPR54 and LHCGR encoding LH receptor have been associated with precocious puberty by the stimulatory effect of kisspeptin on gonadotropin synthesis and dysregulating Leydig cell function, respectively [25,28]. Meanwhile, the pathophysiology of GDPP induced by the GoF mutation of the *TSHR* mutation is poorly understood, and a prolonged hyperthyroid state seemed mostly responsible for early pubertal progression.

According to the latest guideline, appropriate ablative therapy remains the only optimal treatment for NAH to arrest persistent hyperthyroidism and avoid its relapses and consequences, since there have been no randomized controlled trials assessing this condition owing to the rarity of the condition [6]. This could be the first case of NAH in a patient whose hyperthyroidism was well controlled under an antithyroid drug while the thyroid glands were spared. However, thyroidectomy followed by radioiodine treatment seems inevitable after confronting diverse extrathyroidal manifestations, and it is challenging to anticipate further events without fully understanding the nature of the GoF mutation of the *TSHR*. Fortunately, in this case report, the euthyroid state has been achieved and maintained while titrating the methimazole dosage; therefore, a surgical intervention may safely be delayed under close monitoring. In conclusion, this case report describes the case of a boy with persistent sporadic NAH who has undergone diverse extrathyroidal manifestations. Further investigation is warranted to clarify the roles of the functional mutations of TSHR by investigating the correlation between G protein-dependent signaling properties and clinical phenotypes associated with persistent hyperthyroidism; therefore, novel therapies could be provided for numerous conditions caused by NAH.

## Figures and Tables

**Table 1:** Thyroid functions at each extrathyroidal manifestation.

Thyroid Function (Units) (Reference Range)	Ventriculomegaly	Bladder Stone	GDPP
T3 (ng/mL) (0.94–2.41)	4.00	1.58	1.93
fT4 (ng/dL) (0.80–2.00)	3.76	1.40	0.94
TSH (mIU/L) (0.60–8.00)	0.06	0.09	0.01

fT4: free thyroxine; GDPP: gonadotropin-dependent precocious puberty; T3: total triiodothyronine; TSH: thyrotropin.

## Data Availability

No new data were created or analyzed in this study. Data sharing is not applicable to this article.
